# A Rare Entity in Children: Two Cases of Post-traumatic Priapism With Spontaneous Resolution

**DOI:** 10.7759/cureus.80128

**Published:** 2025-03-06

**Authors:** Mohammed Alblooshi, Masih Abdul Kader, Mustafa Hamchou

**Affiliations:** 1 Department of Pediatric Surgery, Tawam Hospital, Al Ain, ARE; 2 Department of Pediatric Surgery and Urology, Al Jalila Children's Hospital, Dubai, ARE

**Keywords:** case report, conservative management, non-ischemic priapism, pediatric priapism, trauma

## Abstract

This report presents two pediatric cases of post‐traumatic high‐flow priapism managed using a conservative approach. The first case involved a six-year-old boy who developed a persistent, painful penile erection three days after sustaining perineal trauma from a seesaw incident. Color Doppler ultrasonography revealed an arteriovenous fistula with a localized hematoma. The second case involved a five-year-old boy who presented with a persistent, painless erection following a bicycle-related groin injury; Doppler ultrasonography confirmed a high-flow arteriocavernosal fistula. Both patients were managed with intermittent penile shaft compression administered in two‑hour cycles (compression followed by release, with immediate decompression upon discomfort). Follow-up duplex ultrasonography at two weeks confirmed the resolution of the fistula, and both patients remained asymptomatic for at least three months. These cases illustrate that a conservative management strategy can serve as an effective alternative to invasive interventions in pediatric post-traumatic high-flow priapism.

## Introduction

Priapism is defined as a prolonged penile erection lasting more than four hours without sexual stimulation [1]. Although it is rare in the pediatric population, with the majority of cases linked to hematological disorders such as sickle cell disease, trauma-induced high-flow priapism represents an uncommon clinical entity [2]. In these cases, a blunt injury to the perineum or penile shaft can disrupt the integrity of the cavernosal arterial wall, leading to the formation of an arteriocavernosal fistula that permits unregulated arterial inflow into the corpora cavernosa. Unlike low-flow (ischemic) priapism, the high-flow variant is typically painless and exhibits distinct ultrasonographic features, including turbulent blood flow and the arterialization of the venous channels [3]. Advances in diagnostic imaging, particularly color Doppler ultrasonography, have greatly enhanced the ability to differentiate between these subtypes [4]. Moreover, while invasive interventions such as selective arterial embolization have been employed in adult populations, concerns regarding potential long-term sequelae on erectile function in children have prompted the exploration of less invasive strategies [5]. Consequently, conservative management methods, such as intermittent penile compression, have emerged as viable alternatives. The objective of this report is to present two pediatric cases of post-traumatic high-flow priapism managed successfully with a conservative approach, thereby mitigating the risks associated with more invasive treatments.

## Case presentation

Case 1

**Figure 1:**
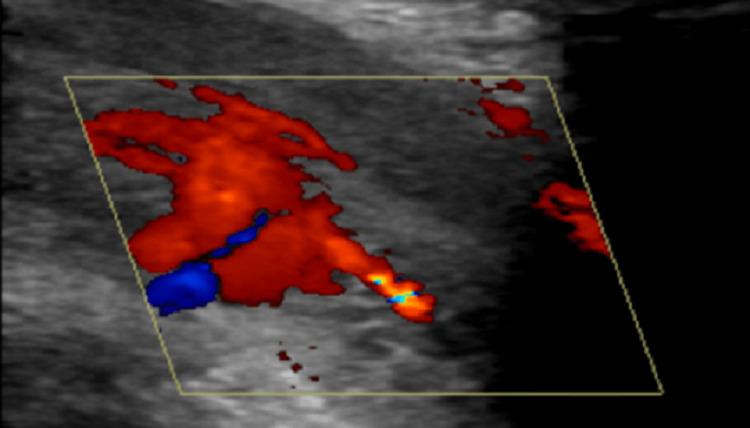
Color Doppler ultrasound image from case 1 demonstrating an arteriovenous fistula with an adjacent hematoma in the right upper cavernosa

A six-year-old boy presented with a three-day history of painful penile swelling following perineal trauma sustained while playing on a seesaw. He had no significant past medical or hematological history. On physical examination, his penile shaft was in a state of persistent erection without discoloration or marked swelling; however, mild tenderness was elicited on palpation. A color Doppler ultrasound examination was performed to assess the vascular flow, which revealed an arteriovenous fistula with evidence of a localized hematoma in the right upper cavernosa adjacent to the penile base, along with the arterialization of the venous flow (Figure 1).

**Figure 2:**
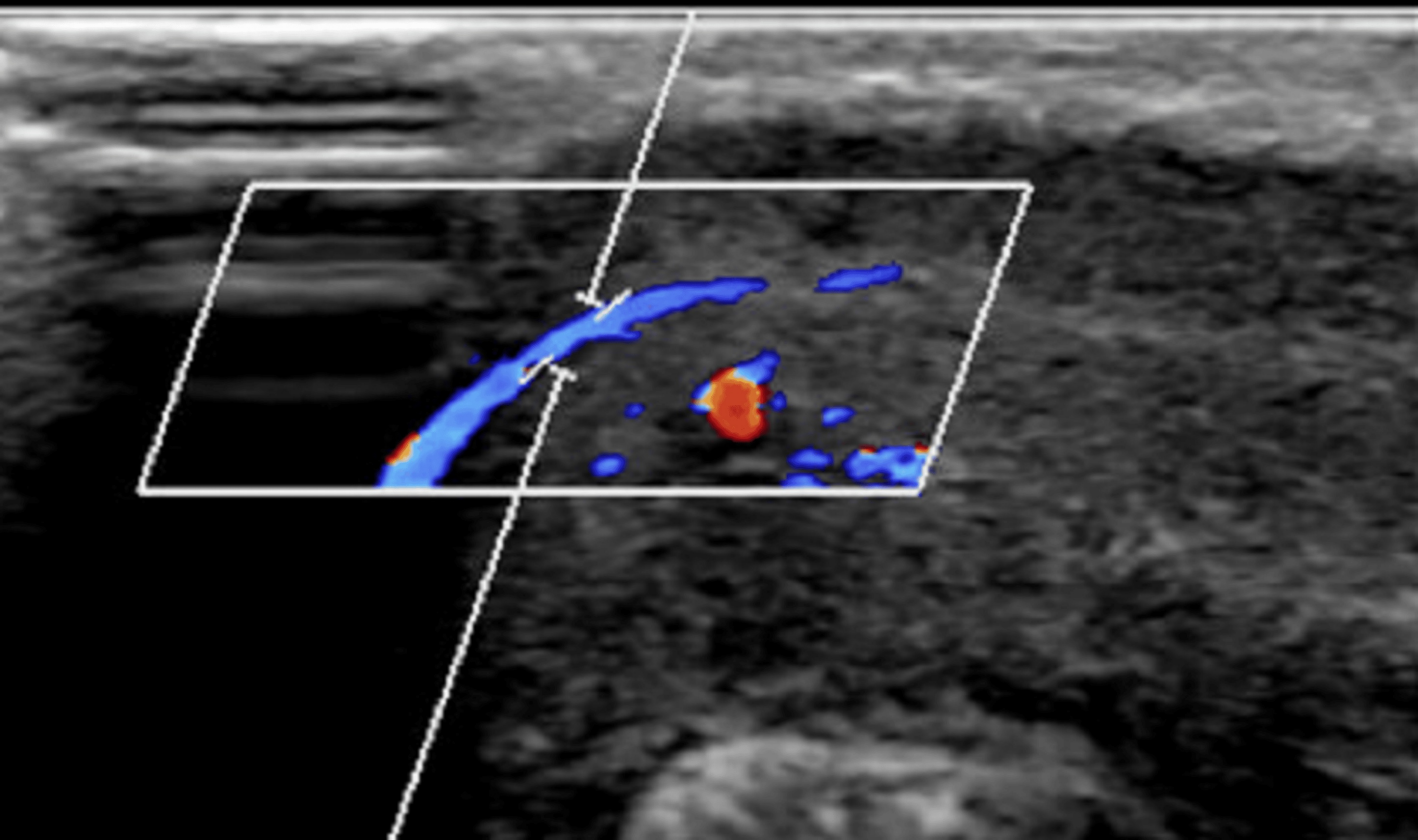
Follow-up duplex ultrasound image at two weeks The previously observed turbulent, high-velocity flow in the right corpora cavernosa has completely resolved. Note that the blue color typically represents venous flow, the red color indicates arterial flow, and the absence of mixed turbulent flow at the injury site confirms the closure of the arteriocavernosal fistula

Following the initial administration of analgesia and patient reassurance, conservative management was initiated with intermittent penile shaft compression. The compression regimen consisted of two-hour periods of applied pressure followed by two-hour intervals of release, with adjustments made based on the patient's discomfort. A consultation with the interventional radiology team confirmed that embolization carried risks such as potential penile tissue damage and the compromise of future erectile function, thus reinforcing the decision to proceed with conservative management. A repeat duplex ultrasound at two weeks demonstrated the complete resolution of the fistula (Figure 2), and the patient remained symptom-free with no recurrence at a three-month follow-up.

Two weeks after presentation, the focal corpora cavernosa hematoma at the mid to distal penile shaft was almost completely resolved, leaving only a small defect or scar. No evidence of loculated collection or echogenic lesions was observed within the corpora cavernosa or tunica albuginea, and the turbulent high vascular flow previously detected at the site of trauma was absent.

Case 2

**Figure 3:**
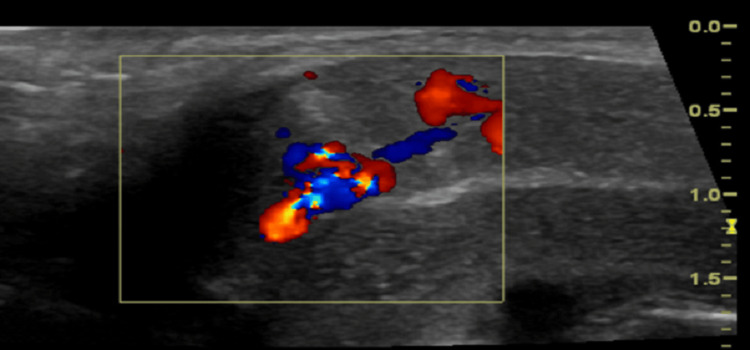
Doppler ultrasound image from case 2 showing turbulent, high-velocity arterial flow consistent with an arteriocavernosal fistula in the right corpora cavernosa

A five-year-old boy presented with a five-day history of persistent, painless penile erection following a bicycle accident that resulted in groin trauma seven days prior to presentation. Initial evaluation at a private clinic had documented corpora swelling and localized tenderness, although priapism was not observed at that time. On arrival at our facility, the patient exhibited an overt penile erection accompanied by gross swelling and mild bruising, with tenderness noted upon the palpation of the penile shaft. An initial Doppler ultrasound performed at the referring clinic suggested a right-sided corpora injury with the possibility of an arteriovenous fistula. A subsequent repeat Doppler ultrasound at our institution confirmed the presence of turbulent, high-velocity blood flow within the right corpora cavernosa, findings consistent with an arteriocavernosal fistula (Figure 3).

**Figure 4:**
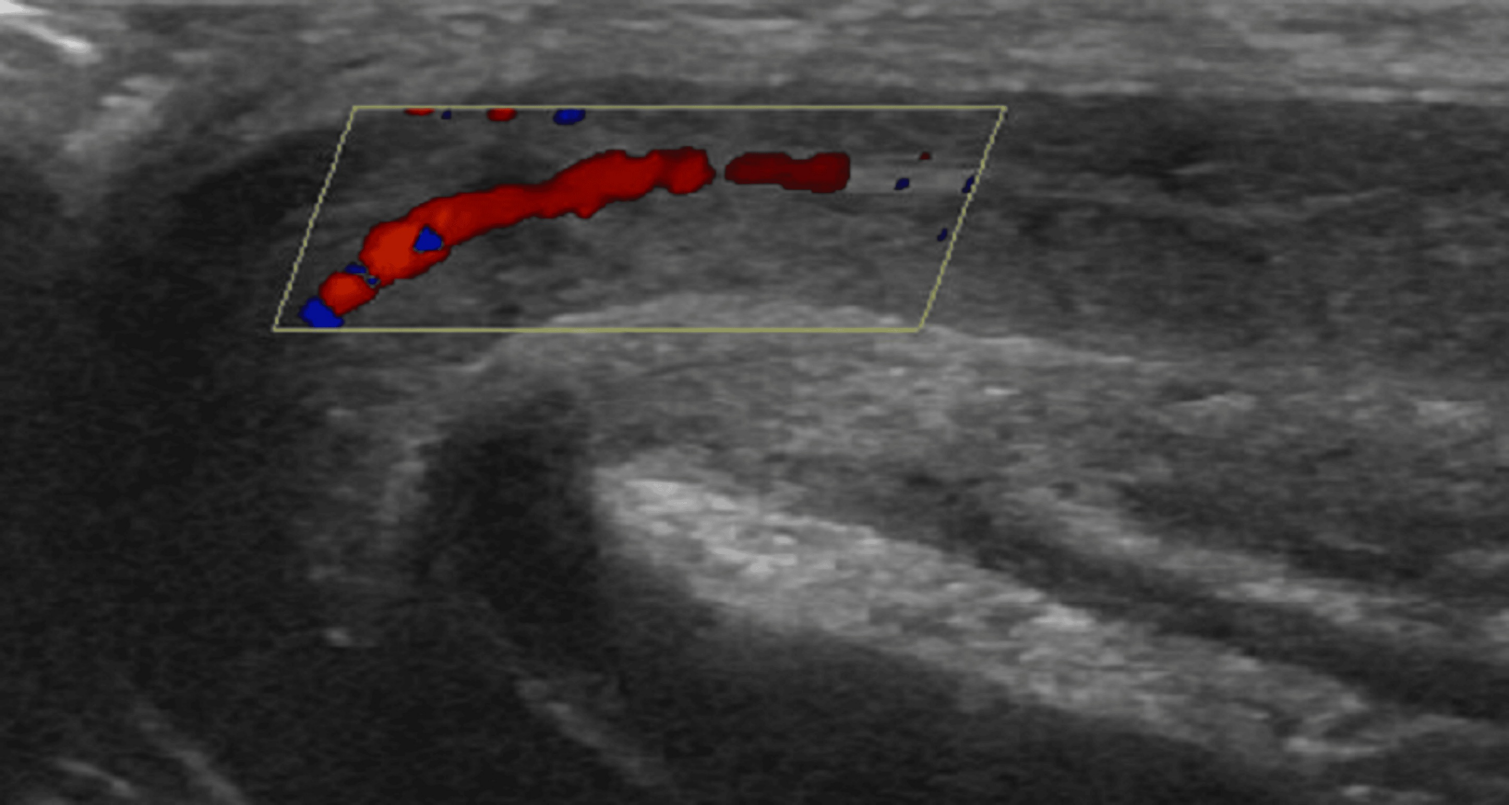
Follow-up duplex ultrasound image at two weeks The observed turbulent, high-velocity flow in the right corpora cavernosa has resolved. Red typically represents arterial flow, and blue indicates venous flow. By six weeks post injury, normal cavernosal artery flow and velocity were observed at the mid to distal shaft, with a small focal heterogeneous area consistent with post-traumatic sequelae

The patient was managed using the same conservative strategy of intermittent penile compression as described in case 1, without the need for any pharmacological or invasive interventions. Supportive measures and pain management were provided as required, and follow-up Doppler imaging after two weeks confirmed the resolution of the abnormal vascular flow (Figure 4). The patient's erection subsided completely, and he remained asymptomatic throughout a three-month follow-up period.

## Discussion

High‐flow priapism in the pediatric population is an exceedingly rare phenomenon, with trauma-induced cases representing only a small fraction of overall presentations. Pediatric priapism itself is uncommon, and while the majority of cases are ischemic, often related to conditions such as sickle cell disease, the non-ischemic, high-flow variant resulting from blunt trauma has been reported in only a few instances [2]. The underlying pathophysiology involves damage to the cavernous arterial system with the subsequent formation of an arteriocavernosal fistula. This abnormal vascular communication allows unregulated arterial blood to continuously fill the corpora cavernosa, leading to a persistent erection that is typically painless. The distinct clinical and ultrasonographic features of high-flow priapism necessitate prompt recognition and differentiation from the ischemic type, which requires more emergent intervention.

The pathogenesis of trauma-induced high-flow priapism is attributed to mechanical injury that disrupts the integrity of the cavernosal arterial wall. Blunt trauma to the perineum or penile shaft can result in the formation of a localized fistulous connection between the cavernosal artery and the erectile tissue [6]. Color Doppler ultrasonography plays a critical role in this setting by not only confirming the diagnosis but also delineating the extent of the vascular injury. This imaging modality demonstrates turbulent, high-velocity flow within the affected corpora and is particularly beneficial in the pediatric population where minimizing invasive procedures and radiation exposure is paramount. Conservative management using intermittent penile compression has emerged as an effective first-line treatment option for high-flow priapism. This method aims to mechanically reduce arterial inflow, thereby facilitating the spontaneous closure of the fistula. In contrast to selective arterial embolization, which, although effective, carries risks such as penile tissue damage and potential compromise of future erectile function, intermittent compression is noninvasive and has shown excellent outcomes when applied appropriately [7]. Our protocol of two-hour cycles of compression followed by two-hour intervals of rest, with modifications for patient comfort, has been supported by similar successful strategies reported in the literature [8,9]. This approach underscores the value of a multidisciplinary strategy that includes input from pediatric surgeons, urologists, and interventional radiologists.

Long-term follow-up is essential to ensure that conservative management has resulted in sustained resolution and to monitor for any late complications. In our cases, repeat duplex ultrasonography at two weeks confirmed the complete resolution of the fistula, and both patients remained asymptomatic at a minimum follow-up of three months. These favorable outcomes are consistent with previous reports and suggest that early identification and conservative treatment can preserve erectile function while avoiding the risks associated with invasive interventions. The success of our management further emphasizes the importance of individualized patient care and the maintenance of a high index of suspicion for traumatic priapism in pediatric patients.

Our report has several strong points. It represents a very rare case of post-traumatic high-flow priapism in a pediatric patient and also details the conservative management applied with minimal risk of complications from invasive procedures. A multidisciplinary approach was conducted, including pediatric surgery, urology, and interventional radiology, which further strengthens the early, tailored management of these few cases for clinical benefit. However, we acknowledge several limitations: primarily, this report is based on only two cases, which may not be fully representative of the wider pediatric population with this condition. Moreover, even though the outcomes in the short term are quite encouraging, the follow-up period is relatively short. Longer follow-ups would be required to assess the long-term efficacy and possible late complications of the conservative management approach. Larger-cohort and longer-duration prospective studies are indicated to further confirm these findings.

In summary, these cases contribute valuable evidence to the literature supporting the conservative management of trauma-induced high-flow priapism in children. The application of intermittent penile compression, guided by meticulous Doppler ultrasound evaluation, provides a safe, effective alternative to invasive treatments. Early diagnosis and tailored management are critical to preventing long-term complications and preserving future erectile function.

## Conclusions

High-flow priapism in the pediatric population is an extremely rare condition, often resulting from trauma and leading to the formation of an arteriovenous fistula. This condition differs from the typical adult presentation, where invasive treatments such as embolization, epinephrine administration, and surgery are commonly used. In pediatric cases, however, conservative management with intermittent penile compression has proven to be an effective and less invasive approach. We advocate for conservative management, including compression and close follow-up with ultrasound to confirm fistula closure. Early recognition and tailored management, as demonstrated in these cases, can result in complete resolution without the complications associated with invasive interventions. Emphasizing the rarity of this condition in children and the distinct management strategy compared to adults is crucial for advancing pediatric care and improving outcomes.​
